# Percutaneous nephroscopy combined with ultrasound-guided negative-pressure suction for the treatment of perinephric abscess: a case series

**DOI:** 10.1186/s12894-022-01091-8

**Published:** 2022-09-03

**Authors:** Enhui Li, Junhui Hong, Mi Zhou, Yuelong Zhang, Xiang He, Dahong Zhang, Weiwen Yu

**Affiliations:** 1 Urology and Nephrology Center, Department of Urology, Zhejiang Provincial People’s Hospital, Affiliated People’s Hospital, Hangzhou Medical College, Hangzhou, Zhejiang China; 2 Department of Urology, Tonglu Hospital of Traditional Chinese Medicine, Hangzhou, Zhejiang China

**Keywords:** Perinephric abscess, Drainage, Percutaneous nephroscopy, Ultrasound guidance, Negative pressure suction, Case series

## Abstract

**Background:**

Drainage is indicated in many patients with a perinephric abscess (PA). Surgical drainage is associated with trauma and slow recovery, while percutaneous drainage can be ineffective in some patients. We report on 11 patients with PA treated by percutaneous nephroscopy combined with ultrasound-guided negative-pressure suction under local anesthesia.

**Methods:**

This case series included 11 PA patients operated on from January 2013 to June 2020. All patients received percutaneous nephroscopy combined with ultrasound-guided negative-pressure suction. Data, including operation time, volume of intraoperative blood loss, volume of intraoperative pus suction, time of postoperative drainage tube indwelling, time to restore normal body temperature, length of postoperative hospital stay, and intraoperative and postoperative complications, were collected.

**Results:**

The age of the patients was 59 (53–69) years. Eight, six, two, and two patients had hypertension, type 2 diabetes, rheumatoid arthritis, and renal calculi, respectively. The operations were successful forall11 patients. Eight, two, and one patients required one, two, and three channels, respectively, to clear their abscess. The average operation time was 44 (30–65) min, and intraoperative blood loss was 16 (10–20) ml. The volume of intraoperative pus suction was 280 (200–400) ml, time of postoperative drainage tube indwelling was 8.2 (6–12) days, and time to restoring normal body temperature was 0.8 (0.5–2) days. The average postoperative hospital stay was 9.8 (7–14) days. No severe intraoperative or postoperative complications occurred. The postoperative follow-up time was typically 4.8 (3–8) months, and there were no recurrences.

**Conclusion:**

Percutaneous nephroscopy combined with ultrasound-guided negative-pressure suction might be a feasible method for treating PA.

## Background

A complicated urinary tract infection is an infection of either the upper or lower urinary tract in a patient at an increased risk of treatment failure or complications [1, 2]. Complications can be systemic (e.g., sepsis) or local (e.g., renal abscess, perinephric abscess [PA], and papillary necrosis). PA is formed by the spread of a pyogenic infection into the adipose tissues between the renal capsule and perirenal fascia [3, 4], and the risk factors include diabetes, immunosuppression, pregnancy, neurogenic bladder, nephrolithiasis, indwelling urinary devices, and urinary obstruction [1, 2, 5]. The incidence of renal and PA in people with diabetes is 46 per 100,000 person-year, compared with 11 per 100,000 person-year in non-diabetic controls [6]. In addition, 20%-60% of patients with PA have associated renal calculi [4]. *Escherichia coli* is the most common cause of PA, followed by other *Enterobacteriaceae* (such as *Klebsiella*sp., *Proteus*sp., and *Serratia*sp.), as well as *Pseudomonas* sp. and *Enterococcus*sp. [1, 3, 7–9].

Due to the non-specificity and complexity of PA, delayed diagnosis, and the limitations of the available treatments, the mortality rate of patients with PA used to be as high as 40–50% [9].

With the continuous advancement of medical techniques, the diagnosis of PA is no longer difficult, and there are more choices regarding treatment. Conventional therapy includes antibiotics and control of the infection source, but it cannot control the space-occupying effect of the abscess, and surgery maybe indicated [1, 2, 7]. The failure of conservative treatment is also an indication for surgery [1, 2, 4, 7]. Conventional open PA incision and drainage and the more recent laparoscopic PA incision and drainage both require general anesthesia and involve surgical trauma, and patients recover slowly. Ultrasound- or computed tomography (CT)-guided percutaneous drainage can be performed but sometimes ineffective due to several disadvantages, including incomplete drainage and requiring repeated drainages, especially when treating large septal abscesses with thick pus [4, 10, 11]. Therefore, developing better treatments has become a hotspot for clinicians.

This study aimed to report on the details and outcomes of 11 patients with PA treated by percutaneous nephroscopy combined with ultrasound-guided negative-pressure suction under local anesthesia. The results suggest this novel method could be used for the effective management of PA.

## Methods

### Study design and patients

This retrospective study included all patients with PA treated by percutaneous nephroscopy combined with ultrasound-guided negative-pressure suction under local anesthesia from January 2013 to June 2020 in Zhejiang Provincial People’s Hospital. This study was approved by the Ethics Committee of Zhejiang Provincial People’s Hospital (No. 2021QT030). The requirement for informed consent was waived by the committee due to the retrospective nature of the study.

### Surgical methods

**Fig. 1:**
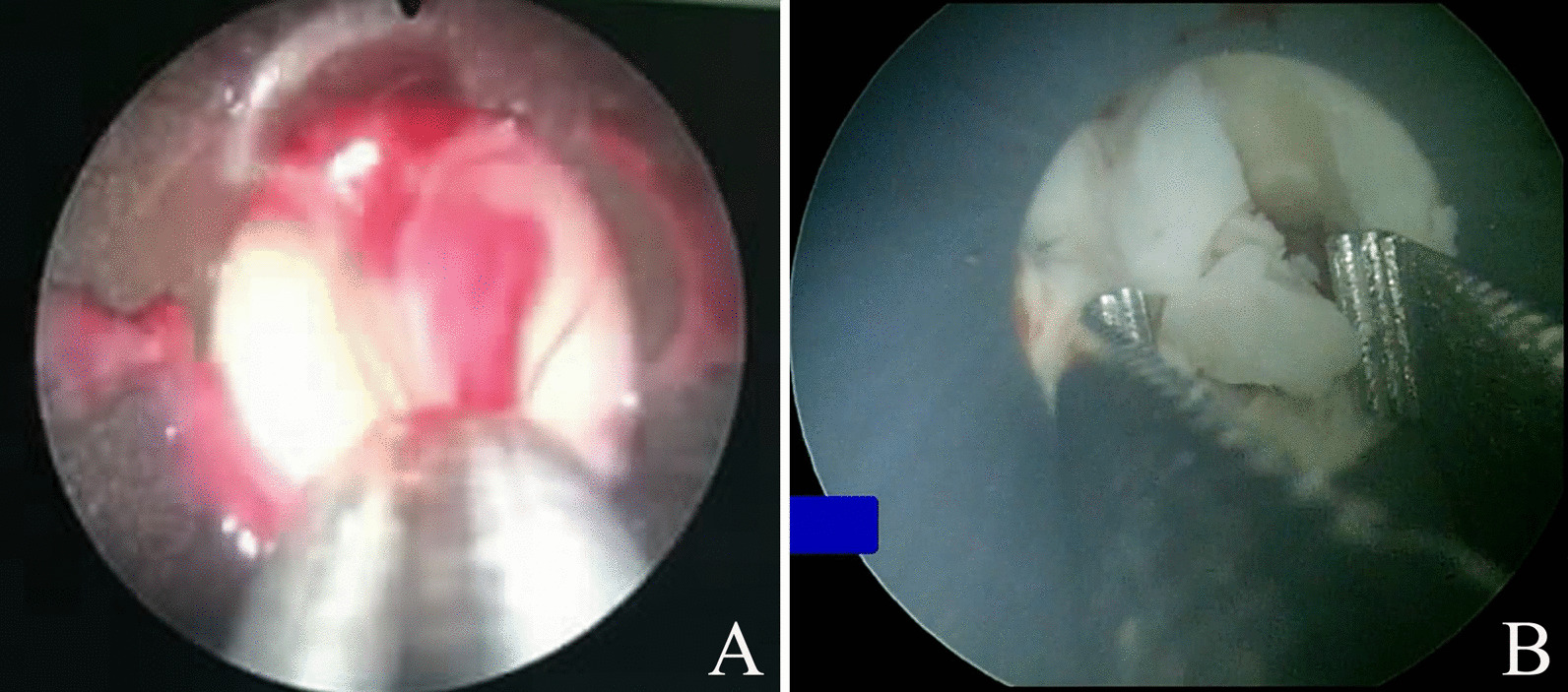
Intraoperative image that shows the percutaneous nephroscopy combined with ultrasound-guided negative-pressure suction for the treatment of perinephric abscess. **A** Negative-pressure suction was used to aspirate the pus. **B** Foreign body forceps were used to remove necrotic tissue

The patients were placed in the prone position with a pad under their waist. A puncture point was selected at the site where the abscess was closest to the body surface while avoiding the major blood vessels and vital organs (e.g., liver and spleen) according to intraoperative ultrasound (BK Ultrasound, Denmark) positioning. Local infiltration anesthesia using 15 ml of 2% lidocaine was performed. An 18 G puncture needle (Create Medic, Japan) was inserted to the clearest sonolucent area of the fluid in the abscess’s center under ultrasound guidance. A small amount of pus was collected for routine bacterial culture and drug sensitivity tests. A fascial dilator (Create Medic, Japan) was used to dilate gradually from 8 to 20 Fr under the guidance of a guidewire (Create Medic, Japan) and then a 20 Fr outer sheath (Create Medic, Japan) was placed. An 18 Fr nephroscope (Richard Wolf, Germany) was inserted through the sheath. Then, negative-pressure suction (EMS, Switzerland) was used to aspirate the pus and tissues completely, and foreign body forceps (Richard Wolf, Germany) were used to remove necrotic tissue in the abscess (Fig. 1). A continuous negative-pressure rinse was performed until the aspirated fluid was clear; then the nephroscope was withdrawn. An 18 Fr drainage tube with multiple lateral holes (Create Medic, Japan) was placed through the sheath, and the incision was sutured to fix the drainage tube. According to the intraoperative ultrasound findings, a second and sometimes third channel was established to further clear septal abscesses, and drainage tubes were placed in each channel. After the surgery, sensitive antibiotics were used for anti-infection therapy. Re-examination by urinary CT was performed when there was no evident pus in the drainage tube. If CT showed that the abscess had disappeared and there was no discomfort after clamping the drainage tube, the patients could remove the drainage tube and be discharged from hospital.

### Data collection

Data, including operation time, volume of intraoperative blood loss, volume of intraoperative pus suction, time of postoperative drainage tube indwelling, time to restore normal body temperature, length of postoperative hospital stay, and intraoperative and postoperative complications were collected. The patients were followed up at the outpatient department, and any recurrences were monitored. Ultrasound examination was performed to monitor recurrences.

### Statistical analysis

Only descriptive statistics were used. Continuous data are summarized using average (range). Categorical data are presented as *n* (%).

## Results

Eleven patients with PA, including three males and eight females, were included in this study. Table 1 presents the characteristics of the patients. The average age of the patients was 59 (53–69) years. All patients had fever and waist pain; two patients had shivering; two had bladder irritation sign; and four had abdominal distension, nausea, and a poor appetite. The abscess was on the left and right side in four and seven patients, respectively. The average maximum diameter of the abscesses was 9.9 (8.1–12.2) cm. Eight, six, two, and two patients had hypertension, type 2 diabetes, rheumatoid arthritis, and renal calculi, respectively. In addition, two patients had secondary infection due to perirenal hematoma after flexible ureteroscopic lithotripsy.

**Table 1:** Characteristics of the patients

Patient No	Age	Sex	Predisposing factors	Presentation	Maximum diameter of the abscess (cm)	Side
1	61	Female	Hypertension and diabetes	Fever, waist pain, nausea, and poor appetite	12.2	Right
2	64	Female	Hypertension, diabetes, and rheumatoid arthritis	Fever, waist pain, shivering, and irritation signs of bladder	9.7	Right
3	57	Male	Perirenal hematoma after flexible ureteroscopic lithotripsy	Fever, waist pain, and abdominal distension	9.6	Left
4	53	Female	Hypertension and diabetes	Fever and waist pain	9.9	Right
5	61	Female	Hypertension and right renal calculus	Fever and waist pain	8.1	Right
6	69	Male	Hypertension and diabetes	Fever, waist pain, and shivering	9.5	Left
7	60	Female	Hypertension and diabetes	Fever, waist pain, nausea, and poor appetite	10.8	Right
8	53	Female	Rheumatoid arthritis and left renal calculus	Fever and waist pain	9.1	Left
9	56	Female	Hypertension and diabetes	Fever, waist pain, and irritation signs of the bladder	12	Right
10	58	Female	Hypertension	Fever, waist pain, and shivering	9.3	Right
11	57	Male	Perirenal hematoma after flexible ureteroscopic lithotripsy	Fever, waist pain, and abdominal distension	8.7	Left

Table 2 presents the characteristics of the operations. Operations for all 11 patients were successful. Eight, two, and one patients required one, two, and three channels, respectively, to clear their abscess. The average operation time was 44 (30–65) min. The average intraoperative blood loss volume was 16 (10–20) ml, and the average volume of intraoperative pus suction was 280 (200–400) ml. The average time of postoperative drainage tube indwelling was 8.2 (6–12) days, and the average time to restore normal body temperature was 0.8 (0.5–2) days. The average postoperative hospital stay was 9.8 (7–14) days. No severe intraoperative or postoperative complications occurred. The average postoperative follow-up time was 4.8 (3–8) months and there were no recurrences.

**Table 2:** Intraoperative and postoperative data

Patient No	Number of channels	Operationtime (min)	Volume of intraoperative blood loss (ml)	Volume of intraoperative pus suction (ml)	Time of drainage tube indwelling (days)	Time of restoring normal body temperature (days)	Postoperative hospital stay (days)	Time of follow-up (months)
1	1	45	10	400	6	0.5	7	3
2	2	65	20	250	12	0.5	14	3
3	1	50	20	250	7	1	8	7
4	1	30	20	300	10	1	13	3
5	1	30	10	200	6	1	7	8
6	1	50	10	200	7	0.5	9	4
7	2	60	20	300	6	0.5	12	5
8	1	30	10	200	11	0.5	8	3
9	3	65	20	380	11	2	12	6
10	1	30	20	300	6	1	8	6
11	1	30	15	300	8	0.5	10	5

## Discussion

Drainage is indicated in many patients with PA. Surgical drainage is associated with trauma and slow recovery, while percutaneous drainage can be ineffective in some patients [4, 10, 11]. Therefore, this case series reports the details of patients with PA who were treated by percutaneous nephroscopy combined with ultrasound-guided negative-pressure suction under local anesthesia. The results suggested that percutaneous nephroscopy combined with ultrasound-guided negative-pressure suction is feasible as a method for treating PA.

The risk factors for PA are diabetes, immunosuppression, pregnancy, neurogenic bladder, nephrolithiasis, indwelling urinary devices, and urinary obstruction [1, 2]. Among the 11 patients in this study, two patients had a clear history of upper respiratory tract infection before onset, considering that bacterial hematogenous infection led to the PA. Two patients had secondary infection due to perirenal hematoma after flexible ureteroscopic lithotripsy. The PA of the other seven patients was mainly considered to be caused by pyelonephritis due to the increase of white blood cells in urine. Which is worthy of our attention, with the wide application of flexible ureteroscopic lithotripsy, many patients have a perirenal hematoma after lithotripsy. The risk factors for perirenal hematoma include high intraoperative perfusion pressure, being female, advanced age, diabetes, hypertension, renal insufficiency, urinary infection, coagulation disorders, the use of antiplatelet drugs, large calculus, long operation time, infectious calculus, and upper urinary tract obstruction [12]. The relatively small perirenal hematoma without secondary infection could be gradually absorbed if there are no clinical symptoms. If the hematoma is followed by secondary infection and PA, the patients will develop a severe fever, and anti-infectious therapy can result in relatively poor effects, and thus, surgical interventions are generally required [10, 12].

It is generally considered that, for PA with diameter of < 3 cm with non-matured and liquified content, sufficient antibiotics should be applied in time as conservative systemic therapy [1, 2, 7]. For PA with diameter of 3–5 cm, on which the effects of simple anti-infectious therapies are not evident, drainage should be performed in time [1, 2, 4, 7]. For PA with diameter of > 5 cm, drainage should be performed as early as possible in addition to anti-infectious therapy [1, 2, 4, 7, 10]. Ultrasound- and CT-guided percutaneous drainage is simple, convenient, minimally invasive, inexpensive, and can be performed under local anesthesia [4, 10, 11]. Nevertheless, this method has several disadvantages, such as incomplete drainage, a high risk of drainage tube obstruction, the inability to drain abscess cavities with incomplete liquefaction or sticky pus, and the requirement for repeated punctures for septal abscesses [4, 10, 11]. Conventional open and laparoscopic PA incision and drainage methods have the advantages of complete drainage, the possibility of rinsing the abscess cavities, and suitability for treating relatively large abscesses, septal abscesses, multiple abscesses, and abscesses with thick pus. Despite this, these methods involve more significant trauma than percutaneous drainage, require general anesthesia, and the patients have a risk of complications, relatively slow recovery, and a longer hospital stay [10, 13]. In addition, laparoscopic surgery should be chosen with caution for patients with a long disease course and severe perinephric adhesions. Peritoneal injury, excessively high CO_2_ pressure, a long operation time, and waist myofascial injuries can spread the infection [13].

Rai et al. [14] and Ng et al. [15] reported that treating PA by percutaneous nephroscopy achieved a satisfactory outcome. In this study, further modifications to this method were explored. An ultrasound-guided perinephric puncture was performed under local anesthesia to establish a percutaneous nephroscopy channel. Then, negative-pressure suction was used to aspirate the pus and tissues, and forceps were used to remove the necrotic tissue. This method is applicable to all PA requiring surgical drainage.

The anesthesia methods for percutaneous nephroscopy are currently general anesthesia and combined spinal-epidural anesthesia [16]. In patients with severe infections, especially with renal insufficiency, the capabilities of water-electrolyte regulation and metabolite excretion are relatively poor, and the risk of general anesthesia is elevated [17, 18]. Pain during percutaneous nephroscopy is mainly from stimulation of the sensory somatic nerves and visceral sensory nerves. As the area of puncture is relatively small, local-infiltration anesthesia is enough to eliminate the pain conducted by the somatic nerves [19]. Advancements in techniques and improvements to equipment have made percutaneous nephroscopy feasible, with the advantages of lower and controllable pressure, a shorter operation time, and providing a safer surgical process. Percutaneous nephroscopy has already been performed under local anesthesia by several groups [19, 20] and could favor patients’ early and rapid recovery.

The establishment of a standard channel for percutaneous nephroscopy under local anesthesia could provide a good visual field while maintaining low perfusion and allowing the rapid and highly efficient clearing of pus by negative-pressure suction. For thick pus that cannot be easily aspirated, suction can be performed after repeated rinsing with normal saline. The locally necrotic tissues, organized blood clots, and pus moss can be aspirated after ultrasound disintegration or using foreign body forceps with nephroscopy. For septal abscess cavities, nephroscopy combined with ultrasound negative-pressure suction can be used to break the relatively thin septa under direct vision before aspirating the pus. For septal abscess cavities that cannot be reached through the first channel, multiple channels can be established under ultrasound guidance according to the sizes and ranges of the abscesses, but wide drainage tubes with multiple lateral holes have to be placed for each channel. For abscesses with liquefaction of necrotic tissues, a small amount of pus can still be drained from the drainage tubes after the operation. Thus, the drainage tube should be maintained in the patient, and sterilized normal saline could be used to rinse if necessary.

We summarize the key points of this operation as follows. First, the center of the abscess should be selected as accurately possible to avoid the pain induced by large angle swings of nephroscopy. Second, low-flow perfusion should be performed to reduce pain and water pressure during rinsing. The puncture must be precise to avoid excessive bleeding, which could lead to an unclear visual field. Third, continuous negative-pressure suction during the operation could reduce the pressure in the abscess cavity, decrease the risk of bacteria and toxin backflow into the blood, and induce fluid exudation, reducing the incidence of postoperative urinary sepsis. Finally, the sites and numbers of channels used for nephroscopy should be appropriately planned under the guidance of ultrasound, and wide drainage tubes with multiple lateral holes should be placed in each channel after the operation to help obtain the best pus-clearing and drainage effects with minimal trauma.

This study had some limitations. The patients were from a single center, and the sample size was small. In addition, only a short follow-up period was included. Prospective studies with a large sample size and long-term follow up are needed to verify our findings.

## Conclusions

In conclusion, this study suggests that percutaneous nephroscopy combined with ultrasound-guided negative-pressure suction might be feasible for treating PA. This method has the advantages of minimal invasion, complete drainage, rapid recovery, and fewer complications. Skillful operators could choose to apply this method.

## Data Availability

The datasets used and/or analysed during the current study are available from the corresponding author on reasonable request.

## Abbreviations

PA: Perinephric abscess
UTI: Urinary tract infection
CT: Computed tomography
